# Application of Reverse Transcriptase –PCR (RT-PCR) for rapid detection of viable *Escherichia coli* in drinking water samples

**DOI:** 10.1186/s40201-015-0177-z

**Published:** 2015-03-20

**Authors:** Neda Molaee, Hamid Abtahi, Mohammad Javad Ghannadzadeh, Masoude Karimi, Ehsanollah Ghaznavi-Rad

**Affiliations:** Department of Microbiology and Immunology, Arak University of Medical sciences, Arak, Iran; Molecular and Medicine Research Center, Arak University of Medical Sciences, Arak, Iran; Department of Environmental Health, Faculty of Health, Arak University of Medical Sciences, Arak, Iran; Department of Medical Microbiology and Immunology, Arak University of Medical sciences, Arak, Iran; Department of Medical Microbiology and Immunology, Faculty of Medicine, Arak University of Medical Sciences, Arak, Iran

**Keywords:** Coliforms, Reverse transcriptase, Drinking water

## Abstract

**Background:**

Polymerase chain reaction (PCR) is preferred to other methods for detecting *Escherichia coli (E. coli*) in water in terms of speed, accuracy and efficiency. False positive result is considered as the major disadvantages of PCR. For this reason, reverse transcriptase-polymerase chain reaction (RT-PCR) can be used to solve this problem. The aim of present study was to determine the efficiency of RT-PCR for rapid detection of viable *Escherichia coli* in drinking water samples and enhance its sensitivity through application of different filter membranes.

**Materials and methods:**

Specific primers were designed for *16S rRNA* and elongation Factor II genes. Different concentrations of bacteria were passed through FHLP and HAWP filters. Then, RT-PCR was performed using 16srRNA and *EF –Tu* primers. Contamination of 10 wells was determined by RT-PCR in Arak city. To evaluate RT-PCR efficiency, the results were compared with most probable number (MPN) method.

**Results:**

RT-PCR is able to detect bacteria in different concentrations. Application of EF II primers reduced false positive results compared to *16S rRNA* primers. The FHLP hydrophobic filters have higher ability to absorb bacteria compared with HAWB hydrophilic filters. So the use of hydrophobic filters will increase the sensitivity of RT-PCR.

**Conclusion:**

RT-PCR shows a higher sensitivity compared to conventional water contamination detection method. Unlike PCR, RT-PCR does not lead to false positive results. The use of *EF-Tu* primers can reduce the incidence of false positive results. Furthermore, hydrophobic filters have a higher ability to absorb bacteria compared to hydrophilic filters.

## Background

The presence of pathogens in drinking water is a major health problem. Therefore, detection and removal of pathogens are considered as main health issues for drinking water. In diagnostic workup, presence of *E.coli* is considered as an indicator of water pollution by wastewater. The conventional methods for detecting pathogens in water such as (most probable number) MPN and water culture are usually costly and time consuming and presumably lack of sufficient accuracy. This is why new methods are expected to detect pathogens in drinking water [1].

Polymerase chain reaction (PCR) could be a good alternative to conventional assays. PCR is a sensitive, accurate and rapid method that its effectiveness has been demonstrated in numerous studies [2]. PCR is able to detect even one bacterium per 100 ml of water [3]. Polymerase chain reaction is a sensitive and accurate method that not only detects pollution, but determines the type of bacteria [4]. However, due to relatively high stability of DNA in the culture medium, presence of dead cells will produce positive result. Some studies showed that the PCR result for bacteria killed by boiling or UV radiation becomes positive. Studies on *E. coli* and *Listeria monocyte genes* showed that the PCR result for autoclaved bacteria is still positive [5].

Due to incidence of false positive results in DNA-based detection procedures, other methods such as initial enrichment before PCR testing are recommended. Although this method increases the detection efficiency of the test for live and dead samples, the duration of the assay will be increased. In addition, in the case where the number of dead bacteria is high, it may lead to false positive results [6].

Due to the low stability of RNA outside the cell, the use of reverse transcriptase polymerase chain reaction (RT-PCR) for detecting RNA is considered as a suitable marker for tracking the living cells. At the same time, the stability of different RNAs varies outside the cell. Davis *et al.,* found that of the four species of nucleic acid, mRNA is the most promising candidate as an indicator of viability in bacteria [7]. Klein and Juneja showed a good correlation between the presence of mRNA and the viability of *L. monocytogenes* when comparing growing cells with those killed by autoclaving [8]. Therefore, this study designed to examine the efficiency of RT-PCR by preparing various concentrations of *E. coli* in water. In addition, the impact of bacterial filters (hydrophilic and hydrophobic) on concentrated water samples and RT-PCR results are studied.

## Materials and methods

PCR primers targeting *16S rRNA* and *EF-Tu* regions of the *E. coli* genome were designed using Primer Select (DNAstar, Inc., Madison, WI) software package. Two sets of primer pairs were designed and tested. The nucleotide sequences of the primers have been shown in Table 1.

**Table 1:** **The sequence and position of the oligonucleotide primers used in the study**

**Gene**	**Forward**	**Reverse**	**Size (bp)**	**Access number**
*16S rRNA*	5′CGA GTG GCG GAC GGG TGA GT3′ (FROM 81)	5′ TCG ACA TCG TTT ACG GCG TGG A3′ (FROM 786)	723	EF620925
*EF-Tu*	5′CGCTGGAAGGCGACGCAGAG 3′ (FROM 1253)	5′CGGAAGTAGAACTGCGGACGGTAG3′ (FROM 1698)	470	X57091

### Water dilutions

To prepare water dilutions, sequential dilutions of *E. coli* bacteria were prepared in 100 ml of sterile water. The McFarland Solution No.1 was used to prepare different bacterial concentrations. For this purpose, the bacterial suspension is adjusted to match the one McFarland turbidity standard.

Then, the suspension was used to prepare sequential dilutions of 8/100 to 1/1600 as listed in Table 2 (two samples for each dilution). To verify the number of bacteria in various concentrations, the bacterial sediment was obtained by centrifuging the whole water at 5000 rpm for 5 min and cultured by pour plate method in nutrient agar medium. Two concentrations were prepared in two series.

**Table 2:** **Coliforms serial dilutions prepared in laboratory**

**Dilutions**	**1600/1**	**1/800**	**1/400**	**1/200**	**1/100**	**2/100**	**4/100**	**8/100**
Number of bacteria	1	1	1	1	1	2	4	8
The volume of water(ml)	1600	800	400	200	100	100	100	100

After preparing water samples with various bacterial concentrations, a series of dilutions were passed through FHLP filter (Millipore, pore diameter of 0.5 microns) and the second series were passed through HAWP filter (Millipore, pore diameter of 0.45 microns). Then, the filters were placed inside sterile micro tubes and 50 ml water containing 0.1% diethylpyrocarbonate (DEPC water) was added. The micro tubes were vortexed vigorously to release bacteria from the filter surface to the fluid.

### RT-PCR

**Figure 1:**
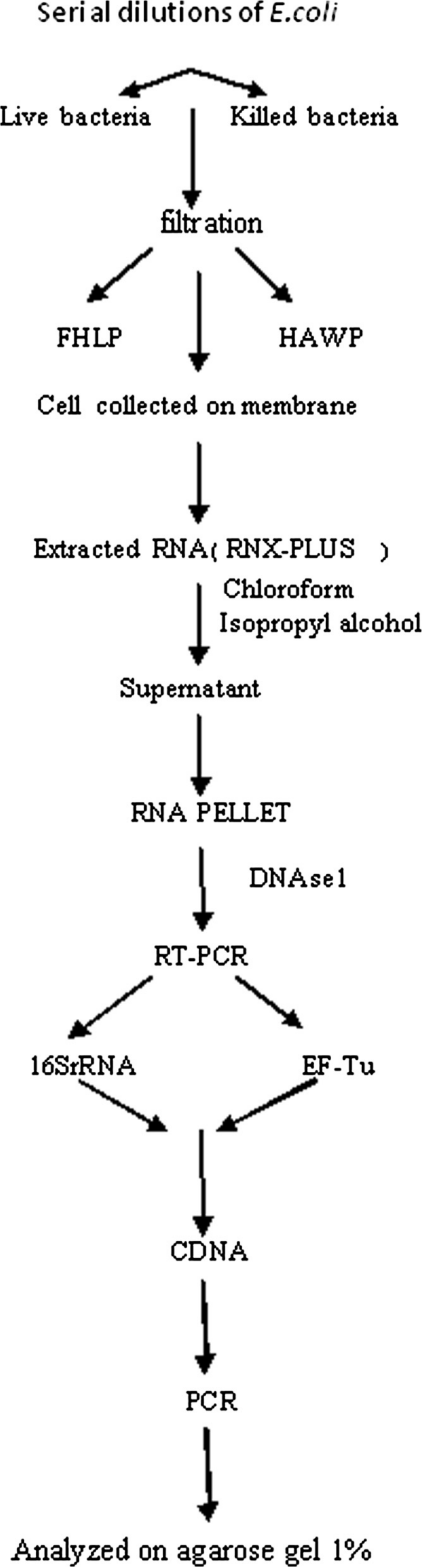
**Flow chart illustrates the RT-PCR procedure for detecting** ***16S rRNA*** **and** ***EF-Tu*** **mRNA of** ***E.coli*** **.**

The bacterial RNA was extracted and purified using the RNX-plus kit (Sinagene, Iran). Deoxyribonuclease I enzyme was used to remove DNA contamination of the purified RNA. To remove the enzyme, 1 μl ethylene diamine tetra-acetic acid (EDTA, 25 Mm) was added for 10 min at 65°C. RT-PCR was performed for *16 s rRNA* and *EF-Tu* under the circumstances described elsewhere [9]. The method is described briefly in Figure 1.

The first phase of PCR is composed of 35 cycles, each consists of three steps including 1-denaturation (at 94°C for 1 min), 2- binding the primers to the DNA template (at 59°C, 1 min for EF-Tu and 1 min for 16SrRNA) and 3- target gene amplification (at 72° for 1 min). After the end of the cycles, the final extension was performed for one cycle at 72 ° C for 5 min.

To evaluate the PCR products, the products were electrophoresed on 1% agarose gel in Tris base- boric acid- EDTA (TBE, pH = 8) buffer solution. To analyze the electrophoresis results, the samples were stained with ethidium bromide solution and visualized by an UV transilluminator apparatus.

**Figure 2:**
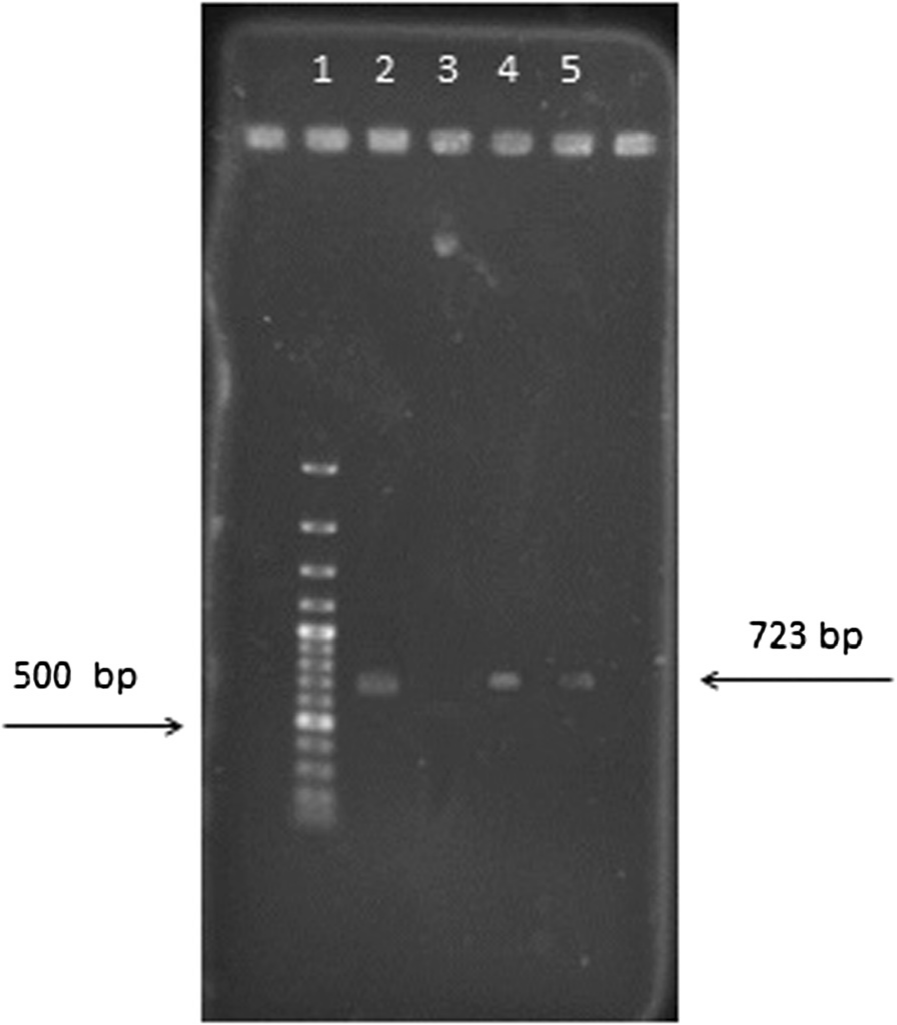
**16SrRNA gene RT-PCR and PCR results : Lane 1: Marker 100 bp, Lane2, a live bacteria RT-PCR, Lane3, death bacteria RT-PCR, Lane 4, a live bacteria PCR, Lane 5, death bacteria PCR.**

To confirm the difference between PCR and RT-PCR in diagnosis of live and death bacteria, control for living and dead bacteria through RT-PCR method and a control of PCR for dead bacteria have been performed in this experiment (Figures 2).

### The stability of *EF-Tu* and *16S rRNA*: mRNA detection in live bacteria

To investigate the stability of RNA in the medium, the bacterial dilutions were killed by chlorine. Then, RNA was extracted and purified at different times (0, 0.5, 1, 2, 3, 4, 6, 10 and 16 h after bacterial death).

To prepare the chlorine solution, 0.1 g of the powdered calcium hypochlorite granules 70% was dissolved in 100 ml water. Then, 30 ml chlorine solution was added to the bacterial dilutions so that the concentration of the residual chlorine was 0.2 to 0.4 mg/l after 30 minutes. After chlorination, the water dilutions were cultured in nutrient agar medium by pour plate method to ensure the death of all bacteria.

After chlorination, series of bacterial dilutions passed through both filters. RT-PCR was performed for genes, *EF-Tu* and *16srRNA* as described before.

### Examination of well water samples by RT-PCR and MPN

To achieve this purpose of the study 10 wells were selected and 250 ml water was collected in sterile containers from each well in Arak city according to standard procedure.

The MPN method was used to examine the consistency of RT-PCR results. The presence of coliform in the samples was evaluated using both methods. In the RT-PCR method, 100 ml water was filtered using HAWP and FHLP filters and then checked for *Escherichia coli*. The MPN test was performed by three-tube method and the number of *E. coli* bacteria was determined based on gas production.

## Results

### Water dilutions

The number of bacteria was confirmed by culturing the water dilutions in agar nutrient medium by pour plate method. The result has been shown in Table 3.

**Table 3:** **The number of colony obtained on in agar nutrient medium by pour plate method**

**Dilution**	**1/1600**	**1/800**	**1/400**	**1/200**	**1/100**	**2/100**	**4/100**	**8/100**
Colony number	1	1	1	1	1	2	4	8

### Water dilutions filtration and RT-PCR

**Figure 3:**
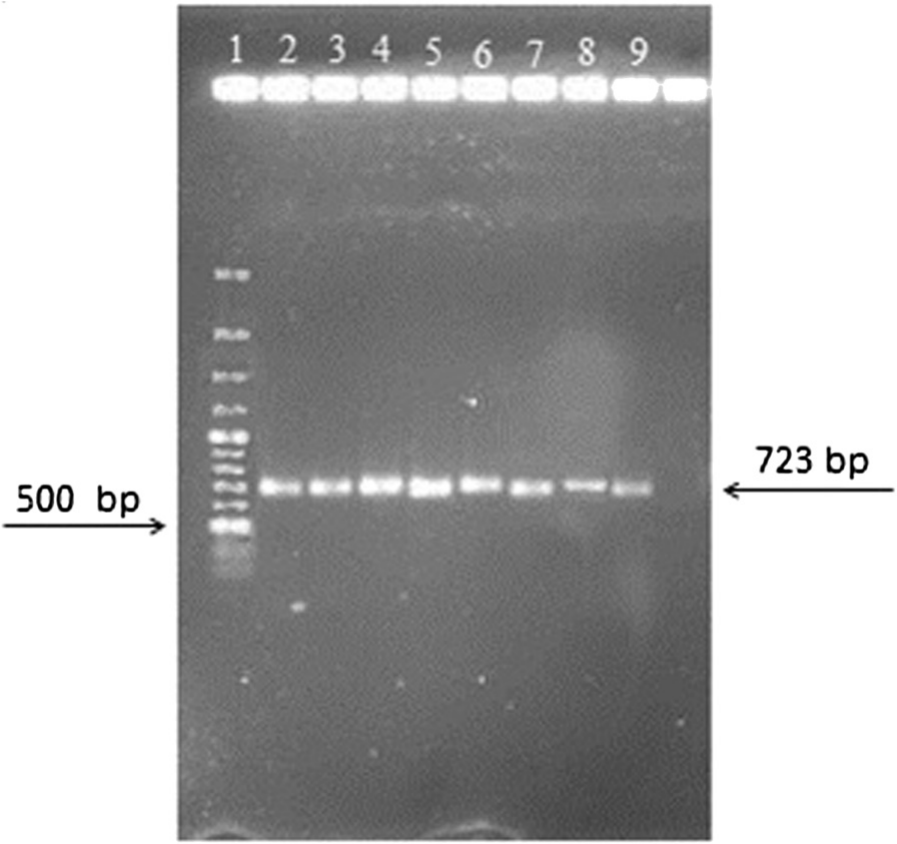
**Dilution of bacterial** ***16SrRNA*** **gene RT-PCR results filtered with FHLP, Lane1, Marker 100 bp, Lane2, 1/1600Dilution, Lane3, 1/800Dilution, Lane 4, 1/400Dilution, Lane 5, 1/200Dilution, Lane 6, 1/100Dilution, Lane 7, 2/100Dilution, Lane 8, 4/100Dilution, Lane 9, 8/100Dilution.**

**Figure 4:**
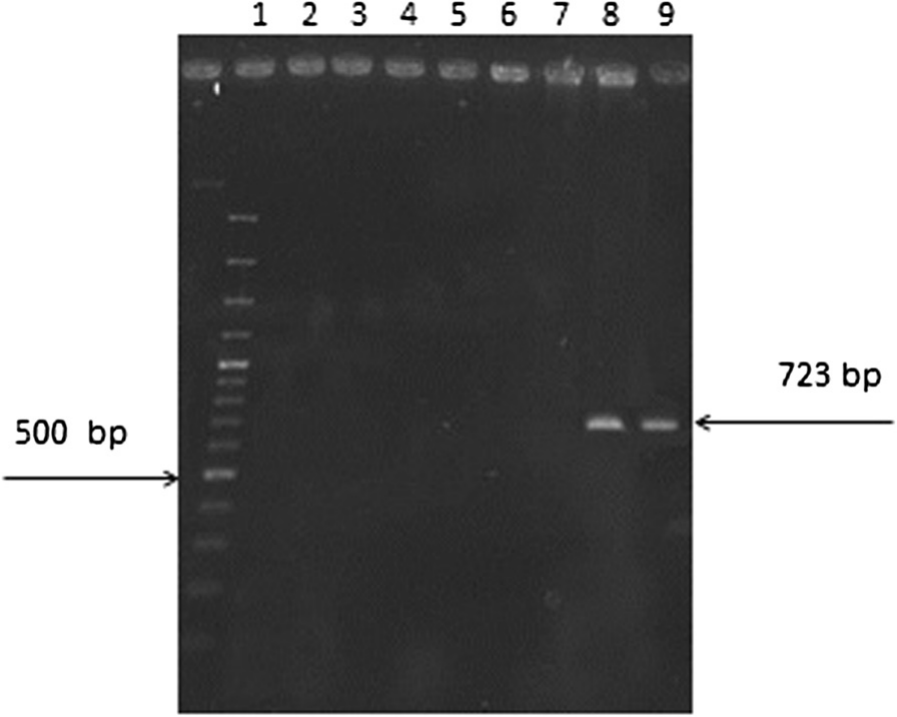
**Dilution of bacterial** ***16SrRNA*** **gene RT-PCR results filtered with HAWP, Lane 1, Marker 100 bp, Lane2,1/1600Dilution, Lane3, 1/800Dilution, Lane 4, 1/400Dilution, Lane 5, 1/200Dilution, Lane 6, 1/100Dilution, Lane 7, 2/100 Dilution, Lane 8, 4/100Dilution, Lane 9, 8/100Dilution.**

**Figure 5:**
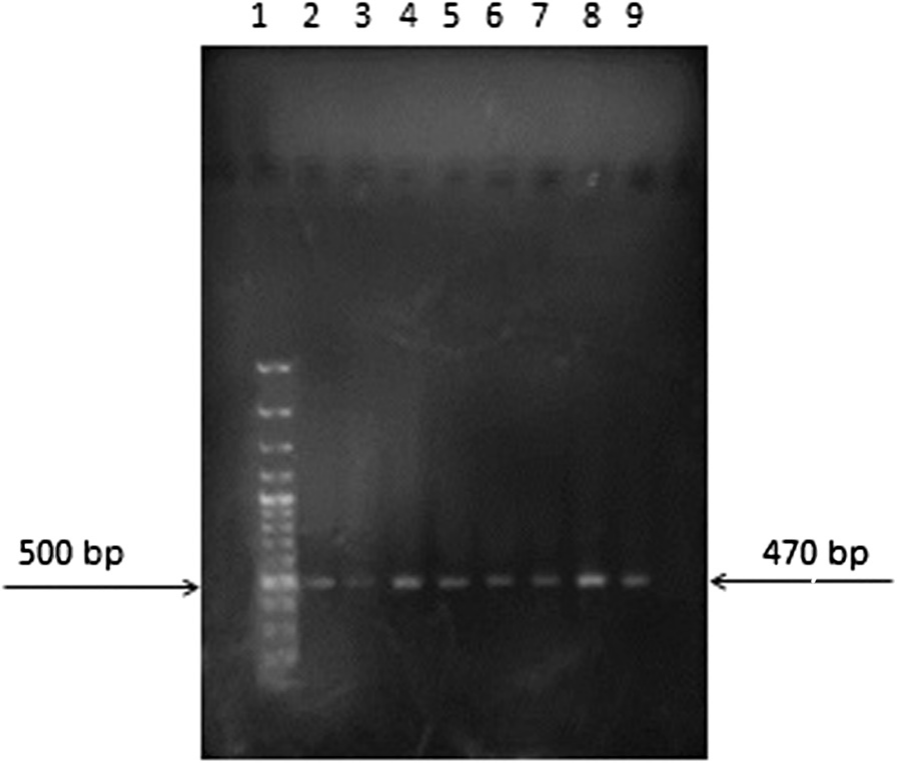
**Dilution of bacterial** ***EF-Tu*** **gene RT-PCR results filtered with FHLP, Lane 1, Marker 100 bp, Lane2, 1/1600 dilution, Lane3, 1/800 dilution, Lane 4, 1/400 dilution, Lane 5, 1/200 dilution, Lane 6, 1/100 dilution, Lane 7, 2/100 Dilution, Lane 8, 4/100 dilution, Lane 9, 8/100 dilution.**

**Figure 6:**
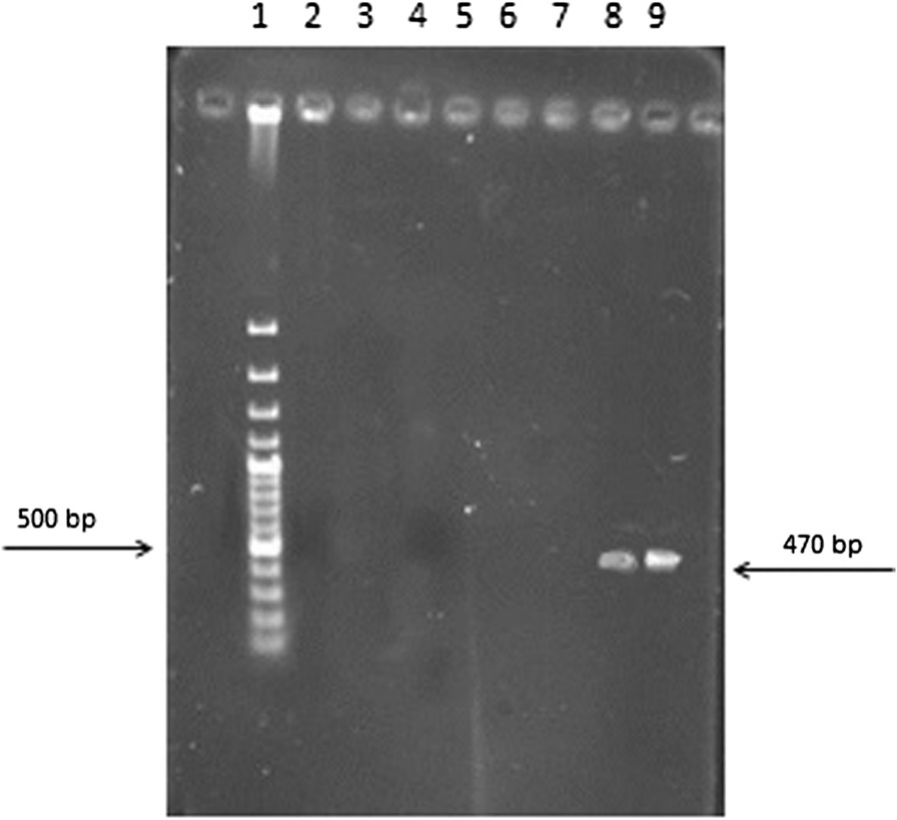
**Dilution of bacterial** ***EF-Tu*** **gene RT-PCR results filtered with HAWP, Lane 1,100 bp ladder, Lane2, 1/1600Dilution,Lane3, 1/800Dilution, Lane 4, 1/400Dilution, Lane 5, 1/200Dilution,Lane 6, 1/100Dilution,Lane 7, 2/100Dilution, Lane 8, 4/100Dilution, Lane 9, 8/100Dilution.**

The result of RT-PCR on water dilutions showed that the RT-PCR results become positive only after filtration with hydrophobic filters (FHLP). When FHLP filters were used, the presence of even one bacterium in 1600 ml water can be detected by RT-PCR. The RT-PCR results for the genes *16S rRNA* (Figures 3 and 4) and *EF-Tu* (Figures 5 and 6) demonstrate the role of FHLP and HAWP filters in the detection of low numbers of bacteria in water samples. The RT-PCR results for all dilutions passed through FHLP showed a band as observed by UV transilluminator.

### The stability of *16SrRNA* and *EF-Tu* after chlorination

No bacteria were found in cultures after chlorination of water dilution samples. Therefore, it was confirmed that bacteria were killed.

Table 4 shows the stability of *16SrRNA* and *EF-Tu* RNAs after the death of bacteria by chlorine. As shown, *EF-Tu* and 16S rRNA remained stable up to 4 h and over 16 h, respectively.

**Table 4:** **Stability of the mRNA (** ***EF-Tu*** **and** ***16S rRNA*** **) in different times was shown**

**Target**	**0 min**	**30 min**	**1 h**	**2 h**	**3 h**	**4 h**	**6 h**	**10 h**	**16 h**
16SrRNA	Y	Y	Y	Y	Y	Y	Y	Y	Y
EF-Tu	Y	Y	Y	Y	Y	Y	N	N	N

Y, Positive RT-PCR. N, négative RT-PCR.NP not performed.

### Comparison of molecular and MPN methods

Table 5 shows the results of MPN and RT-PCR tests for 10 drinking water wells. Comparison of RT-PCR results demonstrates the higher efficiency of RT-PCR method for bacteria detection compared to MPN method. The results of this comparison are given in Table 5.

**Table 5:** **Comparison of RT-PCR result for** ***EF-Tu*** **and** ***16S rRNA*** **gene with the MPN method of 10 wells**

**Number of wells**	**1**	**2**	**3**	**4**	**5**	**6**	**7**	**8**	**9**	**10**
RT-PCR***(*** **16S** ***rRNA)***	-	+	+	+	-	+	+	+	+	+
RT-PCR***(*** ***EF-Tu)***	-	+	+	+		+	+	+	+	+
MPN	-	-	+	-	-	-	-	+	+	-

## Discussion

Due to the importance of water borne diseases, in particular those transmitted by drinking water, the evaluation of water quality in terms of pathogenic microorganisms has a major influence on public health program. The presence of coliforms (especially *E. coli*) in water should be considered as a pollution indicator. Various methods have been designed to detect pathogens in water samples. To approve the water quality in terms of pathogens, the water samples should be exactly checked for the presence of coliforms, especially *Escherichia coli*. Therefore, the presence of coliforms after treatment indicate the water pollution, needs further action. In the case of water contamination with coliforms, the water samples may be contaminated with other pathogens, parasites, viruses, and etc. [10].

There are several problems with viable culture methods (MPN) used for routine monitoring of the bacteriological safety of water supplies, including maintaining the viability of bacteria between the time of collection and enumeration, lack of growth of viable but nonculturable bacteria. Therefore, the use of an ideal method with advantages such as high speed, sensitivity and accuracy, ease of doing and low cost to evaluate a high volume of water samples can be effective in preventing the spread of infectious diseases through water. In addition, detection, separation of most pathogens in contaminated water is difficult, time consuming and often associated with high costs [11]. Although the use of molecular techniques for detecting pathogens is preferred in terms of speed and accuracy, techniques like PCR have some drawbacks. The major disadvantages is that this method is not able to discriminate dead and living bacteria, hence presence of dead bacteria leading to false positive result [6].

Methods that only detect live bacteria must be used to fix this problem. Techniques like RT-PCR can be used to detect live bacteria. In general, RNA-based techniques can prove the presence of living cells only when the target molecule has little stability after cell death. In fact, mRNA molecules are constantly reproduced and increased inside live cells. However, RNA unlike the DNA has a very short half-life, so that RNA content decreases rapidly after the cell death. Therefore, the presence of RNA molecules is a good marker to prove the existence of living cells. Vaitilingom *et al.,* used RT-PCR method and found RNA molecules only in living cells [3]. However, some types of RNA molecules are more stable. For instance, the results of study conducted by Sheridan *et al.,* showed that *16S rRNA* genes remain stable for about 16 h. Therefore, less stable RNA molecules should be sought [12].

The results of the present study showed that the molecular RT-PCR method is more efficient for detecting less stable *EF-Tu*, can reduce the detection time to 4 hours.

Another important point is the higher efficiency of methods like RT-PCR. Various studies suggest the lower sensitivity of RT-PCR than PCR. In this regard, Liu et al. indicated that RT-PCR is not capable of detecting bacteria in suspension with 1 CFU/ml [13]. Thus, hydrophobic filters can be used to concentrate bacteria and increase the sensitivity of RT-PCR to prevent loss of RNA molecules. Bej *et al*., found that hydrophobic filters have a higher ability to absorb bacteria and DNA particles than hydrophilic filters [11]. Our results indicating that the RT-PCR efficiency increases using hydrophobic filter even at low bacterial concentrations. The enhanced efficiency can be attributed to the lack of bacteria loss and thus the presence of bacteria nucleic acids. Although the pore diameter of hydrophilic filter (e.g. HAWP) is less than FHLP filters, the hydrophilic filters are less able to absorb bacteria. HAWP filters only able to isolate the bacteria at dilutions of 4 bacteria in 100 ml water. In other words, RT-PCR is not able to detect bacteria at dilutions less than 4 bacteria using hydrophilic filters.

Beside the ability to detect one bacterium in 100 ml water, the results also indicating that RT-PCR is able to detect a bacterium in higher volumes (up to 1600 ml water) using hydrophobic filters. In addition to the high accuracy and efficiency of the RT-PCR method in water pollution detection, it reduces the time of bacterial detection. The routine MPN test lasts at least 24 h, while this time is reduced to 6 to 7 h using RT-PCR method. Therefore, the test could be started within four hours after sampling to avoid detection of possible contamination with dead bacteria.

The results of polymerase chain reaction (PCR) test in water purification and disinfection systems become false positive due to the stability of DNA molecule. Accordingly, molecular methods like PCR cannot reflect the impact of disinfectants on water pollution index. To resolve this problem, other cell molecules (e.g. RNAs) with relatively low stability such as *EF-Tu* with the stability period of 4 h can be used. Reverse transcriptase polymerase chain reaction (RT-PCR) must be used to determine the presence of RNA in the medium. The detection performance can be enhanced using hydrophobic filters for relatively high volumes of water with low bacterial concentrations.

## Conclusions

Reverse transcriptase polymerase chain reaction (RT-PCR) is an efficient method for detecting the bacterial contamination of water. The detection performance can be enhanced using hydrophobic filters. The detection time is significantly reduced using less stable ribonucleic acids such as *EF-Tu*.
